# A Systemized Approach to Investigate Ca^2+^ Synchronization in Clusters of Human Induced Pluripotent Stem-Cell Derived Cardiomyocytes

**DOI:** 10.3389/fcell.2015.00089

**Published:** 2016-01-13

**Authors:** Aled R. Jones, David H. Edwards, Michael J. Cummins, Alan J. Williams, Christopher H. George

**Affiliations:** Ionic Cell Signalling, School of Medicine, Wales Heart Research Institute, Cardiff UniversityWales, UK

**Keywords:** induced pluripotent stem cells, cardiomyocytes, calcium signaling, synchronization, oscillation, variability

## Abstract

Induced pluripotent stem cell-derived cardiomyocytes (IPS-CM) are considered by many to be the cornerstone of future approaches to repair the diseased heart. However, current methods for producing IPS-CM typically yield highly variable populations with low batch-to-batch reproducibility. The underlying reasons for this are not fully understood. Here we report on a systematized approach to investigate the effect of maturation in embryoid bodies (EB) vs. “on plate” culture on spontaneous activity and regional Ca^2+^ synchronization in IPS-CM clusters. A detailed analysis of the temporal and spatial organization of Ca^2+^ spikes in IPS-CM clusters revealed that the disaggregation of EBs between 0.5 and 2 weeks produced IPS-CM characterized by spontaneous beating and high levels of regional Ca^2+^ synchronization. These phenomena were typically absent in IPS-CM obtained from older EBs (>2 weeks). The maintenance of all spontaneously active IPS-CM clusters under “on plate” culture conditions promoted the progressive reduction in regional Ca^2+^ synchronization and the loss of spontaneous Ca^2+^ spiking. Raising the extracellular [Ca^2+^] surrounding these quiescent IPS-CM clusters from ~0.4 to 1.8 mM unmasked discrete behaviors typified by either (a) long-lasting Ca^2+^ elevation that returned to baseline or (b) persistent, large-amplitude Ca^2+^ oscillations around an increased cytoplasmic [Ca^2+^]. The different responses of IPS-CM to elevated extracellular [Ca^2+^] could be traced back to their routes of derivation. The data point to the possibility of predictably influencing IPS-CM phenotype and response to external activation via defined interventions at early stages in their maturation.

## Introduction

The ability to reprogramme adult human somatic cells into an embryonic-like pluripotent state (Takahashi et al., 2007; Yu et al., 2007; Park et al., 2008) has revolutionized stem cell biology and represents a technological keystone in new approaches for cell replacement therapy and repair. In the setting of cardiac disease, the development of protocols for differentiating induced pluripotent stem cells (IPS) into cardiomyocytes (IPS-CM; Zhang et al., 2009; Zwi et al., 2009) has created the platform for new drug screening approaches (Yokoo et al., 2009; Braam et al., 2010; Dick et al., 2010; Matsa et al., 2011; Silvester and George, 2011; Harris et al., 2013; Liang et al., 2013; Mercola et al., 2013; Sinnecker et al., 2013), frameworks for exploring inherited arrhythmogenic cardiac disorders (Carvajal-Vergara et al., 2010; Moretti et al., 2010; Dambrot et al., 2011; Itzhaki et al., 2011a; Yazawa et al., 2011; Itzhaki et al., 2012; Jung et al., 2012; Siu et al., 2012; Caspi et al., 2013; Lan et al., 2013; Matsa et al., 2014) and early investigations into cardiac repair (Oh et al., 2012; Lalit et al., 2014; Masumoto et al., 2014; Savla et al., 2014).

Although there is vast potential for the use of IPS-CM across a variety of patient-relevant arenas, there are some important obstacles to be overcome. One of the main limitations at present is the high levels of variability in IPS-CM phenotype resulting from the various differentiation processes. Despite methodological advances (Burridge et al., 2011; Mummery et al., 2012), the reproducible production of phenotypically consistent IPS-CM in the massive quantities that are required for “regenerative” approaches remains a huge challenge (Dolnikov et al., 2006; Itzhaki et al., 2011b; Silvester and George, 2011; Li et al., 2013).

The maturation of individual cells in multicellular populations is dependent on complex modes of cell-to-cell interactions via electrical, physical and chemical coupling (Nakamura et al., 2009; Kholodenko et al., 2010; George et al., 2012; Boileau et al., 2015). A critical determinant of cell phenotype and fate is the synchronization of intra- and inter-cellular Ca^2+^ signaling (Nakamura et al., 2009; Kholodenko et al., 2010; Lakatta et al., 2010; George et al., 2012; Thurley et al., 2014; Boileau et al., 2015). Lewis and colleagues recently showed that the functional maturation of embryonic stem cell-derived CMs (eSC-CM) was associated with the enhanced temporal ordering and diminished variability of Ca^2+^ signaling events within and between eSC-CM (Lewis et al., 2015). Deranged Ca^2+^ signaling was a hallmark of eSC-CM phenotypic deterioration and drug-induced cytotoxicity (Lewis et al., 2015).

In this study, we report on a systematic approach to reconcile the method of IPS-CM maturation [in embryoid bodies (EB) vs. “on plate” culture] with the extent of regional Ca^2+^ synchronization in IPS-CM clusters over a 4 week post-differentiation period. The data provide proof-of-concept evidence that IPS-CM phenotype and response to external cues can be directed by controlling very early events in the maturation and culture processes.

## Materials and methods

### Maintenance of iPS and differentiation into embryoid bodies (EB)

iPS derived from the reprogramming of skin fibroblasts obtained from a 56 year-old male were supplied by Chandran and colleagues (Bilican et al., 2012). iPS were maintained in mTESR1 medium (Stem Cell Technologies), on 25 cm^2^ culture surfaces that had been pre-coated with GelTrex (1:400 (v/v) in incomplete Dulbecco's Modified Eagle Medium (Life Technologies) for 45 min at 37°C). iPS were grown on a 3-day cycle and adherent cells were detached in small clumps to preserve the structural integrity of iPS populations (Mitalipova et al., 2005) using ReLeSR (Stem Cell Technologies) and subsequently re-seeded at a ratio of 1:3.

**Figure 1:**
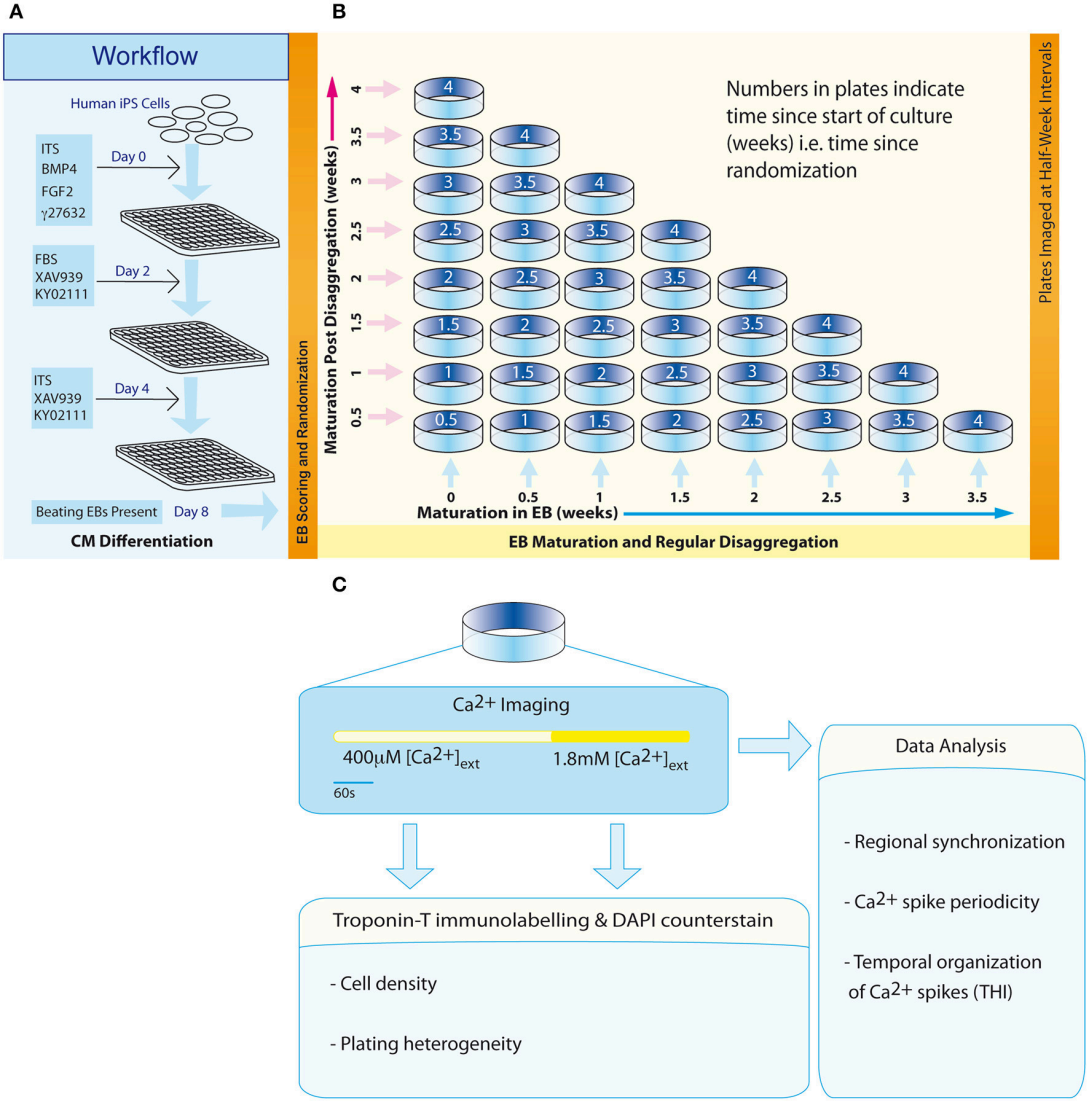
**Workflow and experimental scheme. (A)** An 8-day protocol for production of spontaneously beating EBs. **(B)** The configuration of an experimental matrix to investigate the effect of maturation in EBs vs. post-disaggregation “on plate” culture on regional Ca^2+^ synchronization in IPS-CM clusters. **(C)** Scheme describing the experimental protocol for each plate.

iPS were differentiated into IPS-CM via the formation of EBs using an 8-day protocol adapted from Burridge et al. (2011) that uses three different media formulations with RPMI1640 (supplemented with penicillin / streptomycin [1% (v/v), Life Technologies] as the base media (see Figure 1A). On Day 0 of the differentiation process, iPS maintained as above were detached as single cells using cell dissociation buffer (Life Technologies), counted and transferred into media containing PVA (4 mg/ml), 1-thioglycerol (400 μM, Sigma), insulin-transferrin-selenium supplement (ITS, 1% v/v), chemically-defined lipids (1% v/v, Life Technologies), BMP4 (20 ng/ml, R&D), FGF2 (6 ng/ml, Preprotech), and the rho kinase inhibitor Y27632 (1 μM, Abcam Biochemicals). The cell suspension was plated at a density of 10,000 cells/well in V-bottom 96-well plates to promote forced aggregation (Thermo Scientific, 277143). On Day 2 media was replaced with that containing FBS (20% v/v), 1-thioglycerol (400 μM) and Wnt signaling inhibitors KY02111 (Minami et al., 2012) and XAV939 (Wang et al., 2011; both 10 μM, Tocris). On day 4, iPS were incubated with ITS (1% v/v), lipid (1% v/v), 1-thioglycerol (400 μM), KY02111 (10 μM), and XAV939 (10 μM). EBs were typically obtained on day 4 and were left for 1 day prior to their transfer (to day 5) This protocol avoids the rapid collapse of EB structure reported by others (Burridge et al., 2011). Briefly, on day 5 EBs were separated from the bottom of the well by gentle pipetting and were transferred with all of the supporting media to U-bottomed plates. EBs were subsequently maintained in RPMI1640 containing ITS (1% v/v), 1-thioglycerol (400 μM) and lipids (1% v/v). All plates were maintained at 37°C and 5% CO_2_.

### Analysis of EB structure using confocal laser scanning microscopy (CLSM)

**Figure 2:**
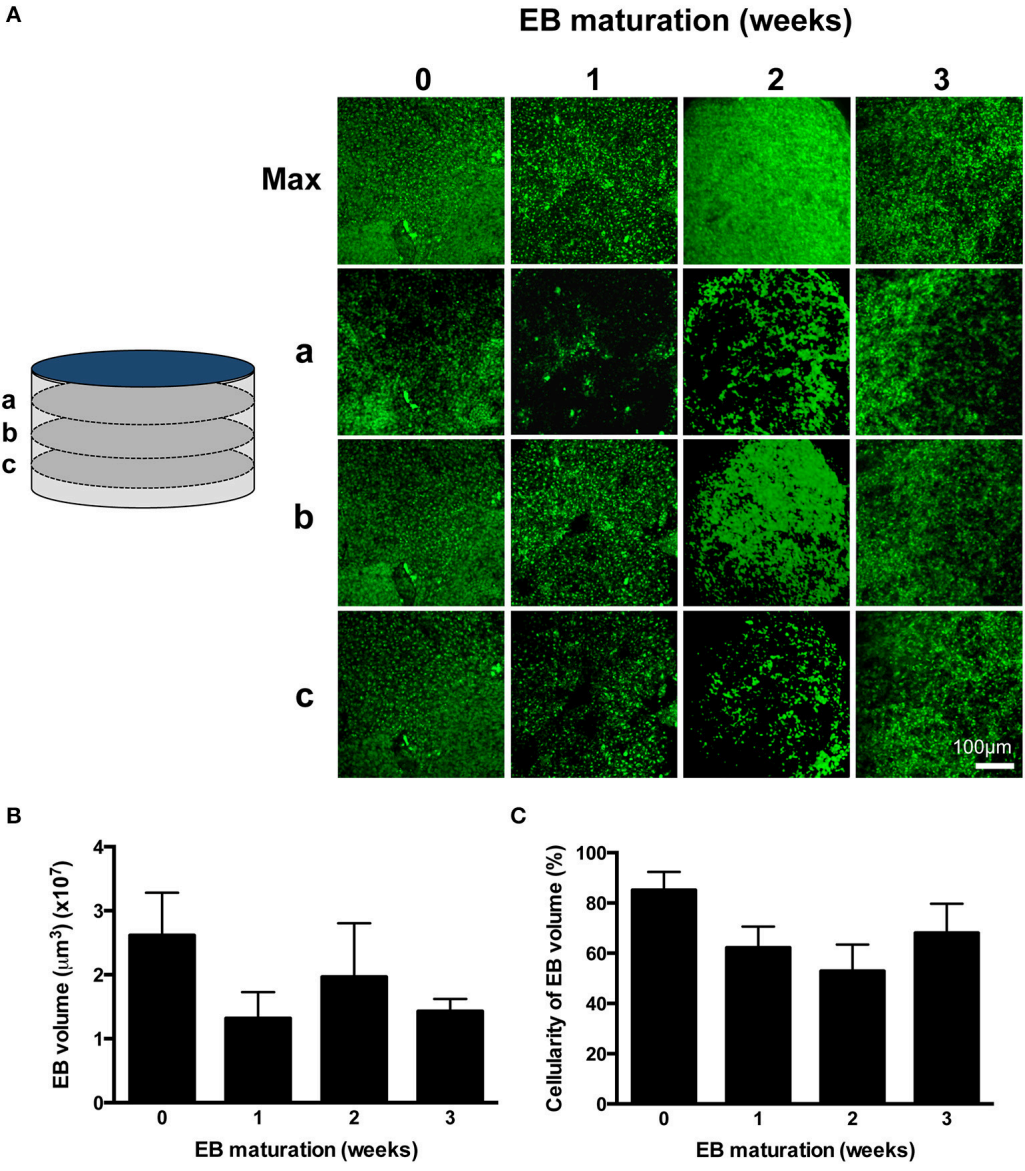
**Profiling EB volume and cellular density. (A)** CLSM z-axis stacks were collected through the entire depth of PI-stained EBs and were reconstructed in maximum projection images (Max). Sections a, b, and c correspond to sections taken at 75% (a), 50% (b), and 25% (c) of the depth of the EB, where 0% is the EB-glass interface. The entire z-axis image sequences were reconstructed for EBs at 0, 1, 2, and 3 weeks and are given in Supplementary Movies 2–5, respectively. **(B)** EB volumes are given as mean ± SE (*n* = 3–6 stacks from separate EBs). **(C)** The proportion of EB volume calculated in **(B)** which was occupied by PI-stained cells was expressed as a percentage. Data are given as mean ± SE (*n* = 3–6 stacks from separate EBs).

EBs were fixed using 4% (v/v) formaldehyde in phosphate buffered saline [PBS, containing (in mM): NaCl (140), KCl (2.7), Na_2_HPO_4_ (10), NaH_2_PO_4_ (2), pH7.4)] for 5–10 min at room temperature, rehydrated in PBS (30 min) and component cells were stained using propidium iodide (PI, 10–25 μM) in PBS which was allowed to equilibrate through fixed EBs. PI labels all post-fixation cellular material (Martin et al., 2005) with spread of the dye through the EB structure facilitated by gap junctions between coupled IPS-CM (George et al., 1998). PI does not accumulate in acellular areas of EBs that result from necrosis (e.g., hypoxic cores; Figure 2A and Supplementary Movies 2–5). Images corresponding to PI fluorescence in orthogonal sections of EBs (i.e., z-axis stacks) were acquired using CLSM and typically 50–80 sections that encompassed the entire depth of the EB (48.38 ± 4.20 μm, *n* = 14 EBs) were acquired. Z-axis image stacks were reconstructed into 360° rotational views of EB structure using Imaris 6.0 (Bitplane). Three-dimensional reconstructions of EBs at 0, 1, 2, and 3 weeks are given in Supplementary Movies 2–5, respectively.

### EB randomization and disaggregation

Three days following the transfer of EBs to U-bottom plates (Day 8, Figure 1A), EBs were scored according to their morphological status and those EBs characterized by well-defined structure were randomly assigned dates for subsequent disaggregation using a computerized random number generator. This randomization time point marked the start of the second phase of the protocol—a 4-week scheme designed to investigate the effect of maturation in EB vs. “on plate” culture [weeks 0 to 4; (Figure 1B)].

Randomized EBs differentiated from the same batch of iPS (Bilican et al., 2012) were disaggregated at half-week intervals using 0.05% trypsin solution (Life Technologies) at 37°C for 2 min. Next, dissociation solution [0.05% trypsin solution (20%), cell dissociation buffer (40%), RPMI (40%)] was added to each well before further incubation for 6 min at 37°C. Well contents were transferred to 1.5 ml centrifugation tubes (2 EBs per tube) and the suspension was centrifuged (400 × g, 3 min). The pellet was re-suspended in RPMI-ITS and then transferred to a 0.1% (w/v) gelatin-coated 14 mm glass-bottomed plate (Mattek Corporation). Plates corresponding to a unique time point in the experimental matrix (Figure 1B) were set up in triplicate and IPS-CM in this “on plate” configuration were maintained in RPMI-ITS. In some experiments, IPS-CM populations in a 0.65 mm^2^ field of view (FOV) were tracked over an initial 48 h period post-plating using time-lapse imaging. Images were acquired every 20 min using an in-incubator microscope fitted with a 10x objective and FOV illumination via a white-light LED (Lumascope, Etaluma) remotely controlled by Lumaview software.

### Ca^2+^ imaging

At half-week intervals throughout the 4-week protocol (Figure 1B, diagonal), plates containing IPS-CM were washed with low [Ca^2+^] Tyrode's solution (LCTS, containing (in mM): NaCl (140), KCl (5.4), CaCl_2_ (0.4), MgCl_2_ (1), HEPES (10), and glucose (10); pH 7.4) and then incubated with LCTS containing fluo4-AM (5 μM, diluted from a freshly prepared 1 mM stock dissolved in DMSO without F-127 pluronic acid; Life Technologies) for 20 min at 37°C, conditions that result in homogeneous loading of cells' cytoplasm and nucleus and not intracellular organelles. LCTS and RPMI1640 both contain free [Ca^2+^] at ~0.4 mM. Imaging of user-defined areas containing IPS-CM clusters was performed using CLSM and a 63X oil-immersion objective (numerical aperture (NA) 1.4) (SP5, Leica Microsystems). Fluorescence and the corresponding brightfield images in B-areas (0.021 mm^2^; **Figure 4A**) were acquired at 512 × 512 pixel resolution at 14.7 frames per second as described (Lewis et al., 2015). After imaging Ca^2+^-dependent fluo4 signals in IPS-CM clusters under basal (non-stimulated) conditions for 330 s, a bolus of CaCl_2_ was added to raise extracellular [Ca^2+^] to ~1.8 mM and imaging was continued for 210 s (Figure 1C). Following Ca^2+^ imaging, plates were fixed using formaldehyde [4% (v/v) in PBS] and IPS-CM nuclei were counter-stained with DAPI (300 nM in PBS, 2 min). Subsequently, five random A-areas of each plate were imaged using a 10X objective [A-areas (0.923 mm^2^), **Figure 4A**].

### Quantifying regional Ca^2+^ synchronization and the temporal organization of Ca^2+^ spikes in IPS-CM clusters

Image series obtained from B-areas (0.021 mm^2^) were subdivided into square grids comprised of 49 identical regions of interest (7 × 7 C-areas, each 0.00043 mm^2^; **Figure 4A**). For each component C-area, the signal intensity traces, corresponding to changes in Ca^2+^-dependent fluo4 fluorescence with time, were exported using LAS-AF Report function (Leica Microsystems) and transferred into Matlab (MathWorks, Inc). Data were de-trended and super-threshold Ca^2+^ signal events (“spikes”) were detected using a custom-made algorithm that calculates local maximum of the first derivative (i.e., Ca^2+^ spikes correspond to the local maximum positive gradient (dF/dt) in the signal where F = fluorescence signal intensity and t = time).

To compute the extent of Ca^2+^ synchronization within a B-area, the temporal organization of Ca^2+^ spikes across the component 49 C-areas was calculated (**Figure 4A**). The perfect temporal co-incidence of Ca^2+^ spikes in one C-area with those in another C-area generates a straight line with a correlation co-efficient (*R*-value) of 1. We considered an *R*-value of ≥ 0.85 as a stringent threshold for robust correlation. For each C-area, the number of the other 48 C-areas that exhibited temporally co-incident Ca^2+^ spikes that met this criterion of an *R*-value ≥ 0.85 was calculated. This process was iterated through all 49 C-areas in the 7 × 7 grid. The extent of regional Ca^2+^ synchronization was thus defined as the proportion of a B-area that exhibited co-incident (synchronized) Ca^2+^ spikes and was expressed as a physical area (μm^2^). Regional Ca^2+^ synchronization in B-areas was visualized by linear interpolation and then assigned a pseudo-color look-up table that spanned the range of values determined.

The temporal organization of Ca^2+^ spikes in C-areas was also used to calculate the mean inter-spike interval (ISI) and the standard deviation of ISI [defined as the temporal heterogeneity index (THI); Lewis et al., 2015; **Figure 6A**]. The numerical value of THI increase as the Ca^2+^ spikes become more temporally disordered (Lewis et al., 2015).

### Assessment of IPS-CM density, plating heterogeneity, and Troponin-T distribution

The density and plating heterogeneity of IPS-CM were calculated from data obtained from five random A-areas on each plate (**Figure 4A**). Cell densities in B-areas (**Figure 4A**) were calculated from the same IPS-CM clusters that had been user-selected for Ca^2+^ imaging. Plating heterogeneity was defined as the standard deviation of regional cell density in A- and B-areas normalized to the corresponding value obtained immediately following the disaggregation of 0-week EBs, which was assigned 1.

To determine the intracellular distribution of troponin-T, IPS-CM were fixed, permeabilized and incubated with a mouse anti-rabbit troponin-T monoclonal IgG which cross reacts with human cardiac troponin-T (clone 13-11, Life Technologies) as described (Lewis et al., 2015). Immunodetection of anti-troponin T antibodies was performed using an Alexa-594 conjugated donkey anti-mouse IgG (Life Technologies) diluted 1:500 (v/v) in PBS. Cell nuclei were counter-stained using DAPI (300 nM in PBS, 2 min).

### Ethics statement

This work used serially passaged iPS lines that had been generated at the MRC Centre for Regenerative Medicine, University of Edinburgh. All protocols were fully compliant with local and national guidelines and legislation.

## Results

### Physical characterization of EBs

The differentiation process adapted from Burridge et al. (2011; Figure 1A) yielded a high proportion of spontaneously-beating EBs [351/480 (73.1%)] and the frequency of beating remained remarkably consistent over 4 weeks (0.24 ± 0.06 Hz, *p* = 0.17, *n* = 23 EBs) (Supplementary Movie 1). The shape of all EBs approximated to flat cylinders (ratio of diameter-to-height = 15.76 ± 2.30, *n* = 9 EBs), the physical dimensions of which did not change over the study. Although there was evidence of a progressive time-dependent change in cellular arrangement within EBs (Figure 2A and Supplementary Movies 2–5), the total EB volume (0.018 ± 0.003 mm^3^, *n* = 9 EBs; Figure 2B) and the volume of the EB that could be ascribed to PI-stained IPS-CM (Figure 2C) remained unchanged (non-parametric ANOVA yielded *p* = 0.57 and *p* = 0.28 for these parameters over the 4-week period, respectively). In three-dimensional reconstructions of EBs we did not observe cavitation or the formation of acellular hypoxic cores (which would not be stained by PI) over the course of the study (Supplementary Movies 2–5).

### EB randomization, disaggregation, and “On Plate” culture of IPS-CM

**Figure 3:**
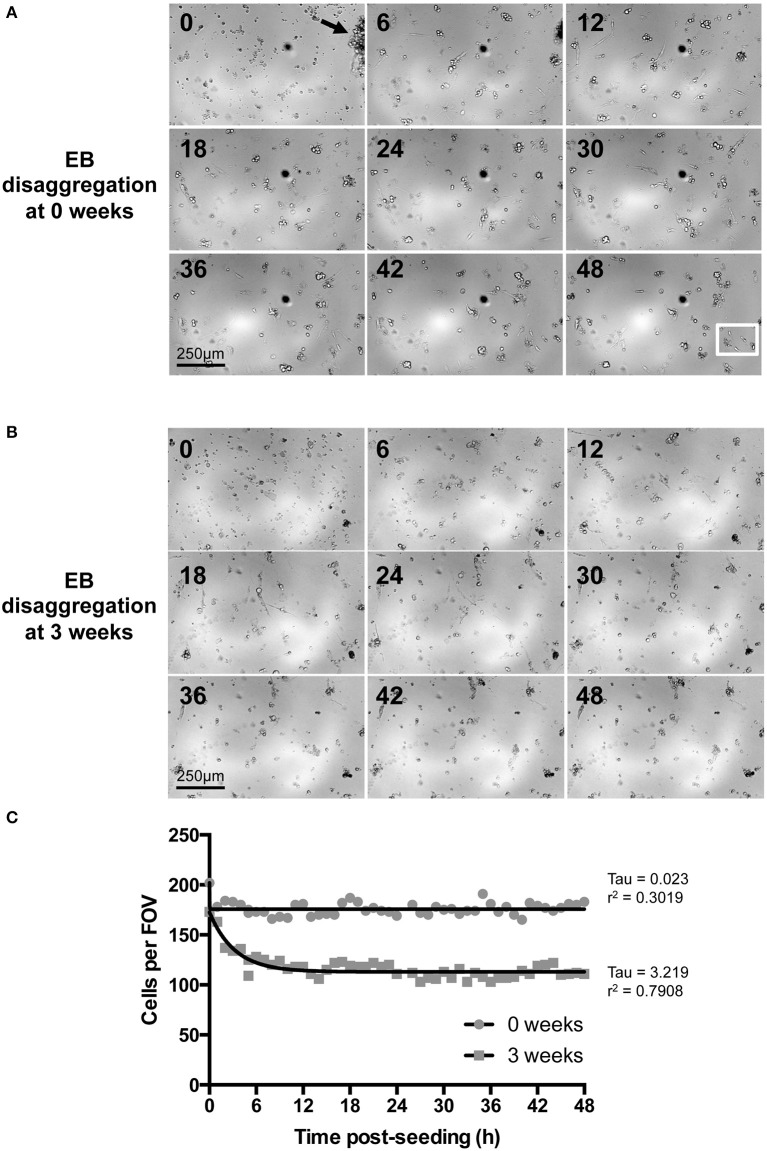
**Time-lapse imaging of IPS-CM following EB disaggregation. (A,B)** Brightfield images of IPS-CM populations taken over a period of 48 h following the disaggregation of 0- and 3-week old EBs [**(A,B)**, respectively]. Images shown correspond to 6 h intervals. The arrow in **(A)** (0 h) shows an IPS-CM cluster which we would typically select for Ca^2+^ imaging in this study. The full image sequence (with images taken every 20 min) corresponding to **(A,B)** are given in Supplementary Movies 6, 7, respectively. The white box in the 48 h panel in **(A)** depicts the area in which we recorded post-disaggregation cell division. The full 48 h time-lapse series of this event is given in Supplementary Movie 8. **(C)** The number of cells per field of view (FOV, 0.625 mm^2^) was counted in each frame of the time series. The net migration of cells out of and into a FOV over the course of 48 h was balanced. Data were fitted with non-linear regression lines of best fit and tau and *r*^2^-values are given. In this example, the number of adherent IPS-CM from 3-week old EBs is reduced by 35.91 ± 0.10 % (mean ± SE) between 12 and 48 h post-seeding when compared to those disaggregated from 0-week old EBs.

**Figure 4:**
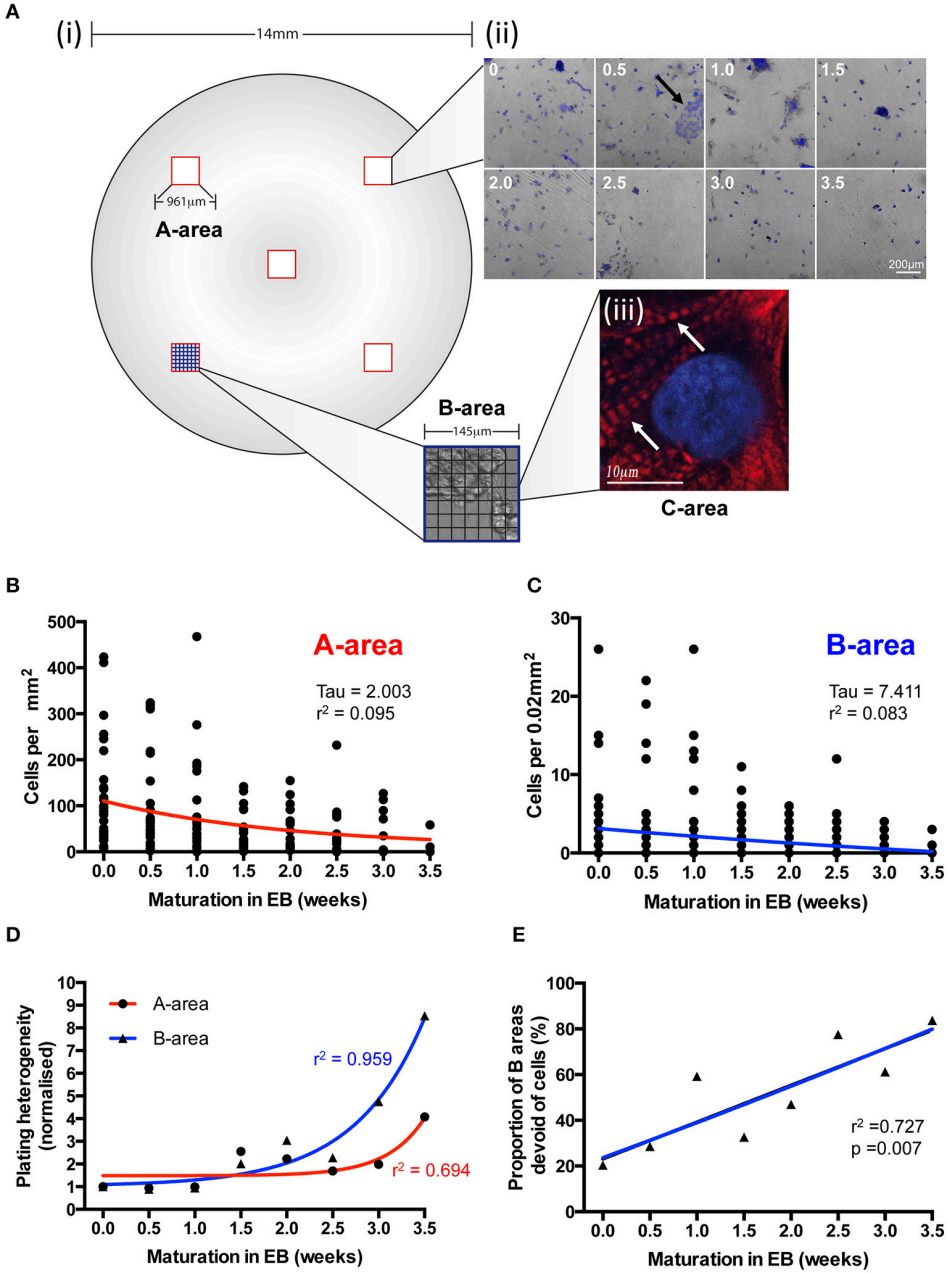
**The effects of EB age on post-disaggregation IPS-CM density and plating heterogeneity. (A) (i,ii)** A grid-based scheme for determining cell density and plating heterogeneity in A- (red), B- (blue), and C- (black) areas of post-EB IPS-CM populations. In this 7 × 7 grid system each B-area corresponds to 1/49th of an A-area and each C-area represents 1/49th of a B-area (see Section Materials and Methods). **(ii)** Representative overlays of DAPI-stained and brightfield images acquired from A-areas of IPS-CM populations following EB disaggregation EBs at 0.5 week intervals. The arrow depicts a typical CM cluster that would be selected for Ca^2+^ image analysis throughout this study. **(iii)** A C-area image of troponin-T immunostaining (red) in an IPS-CM following the disaggregation of a 0.5 week EB maintained “on plate” for a further week. The nucleus is counter-stained with DAPI (blue). Arrow indicates troponin-T striation. **(B,C)** Scatterplots of cellular density in A- and B-areas, respectively, following disaggregation from EBs at 0.5-week intervals. Each point represents the analysis of a single area (*n* = 5–40). Data were fitted with least-squares polynomial function (red and blue lines for A- and B-areas, respectively) and tau and *r*^2^-values are given. **(D)** The plating heterogeneity of IPS-CM was determined from random A-areas (circle) or from B-areas (triangles) across the 4-week protocol. Data are means from *n* = 5–40 areas in each instance and are fitted with least-square polynomial function (A-area, blue; B-area, red) and *r*^2^-values are given. **(E)** The proportion of B-areas that were devoid of cells following post-EB disaggregation and seeding was determined. Data are plotted as means from *n* = 40 A-areas in each instance and were subject to linear regression (blue line) and the *r*^2^-value is given.

Of the spontaneously beating EBs, 330 were selected for subsequent randomization and disaggregation according to the experimental matrix (Figure 1B). Following the disaggregation of 0–3.5 week old EBs via a gentle dissociation protocol designed to retain small CM clusters [see Section Materials and Methods and Figures 3A,B, 4A(ii)], time-lapse imaging revealed that in the first 48 h after seeding onto gelatin-coated glass, the majority of adherent post-EB IPS-CM exhibited dynamic changes in shape and migrated across the culture surface (Figures 3A,B, Supplementary Movies 6, 7). The number of IPS-CM that initially adhered to the gelatin-coated surface (i.e., within the first 6 h of seeding) was reduced following the disaggregation of older EBs (Figure 3C). In all adherent IPS-CM populations the extent of cell death as determined by PI- and Trypan Blue exclusion assays (George et al., 2003) was very low (< 0.5%) and there was no association between the extent of cell death and the age of the dissociated EB (*r*^2^ = 0.03). Likewise, mitotic capacity in post-EB IPS-CM was extremely low (0.01%) and we recorded just one incidence of cell division during the entire study (Figure 3A, white boxed area; Supplementary Movie 8 between 1040 and 1060 min).

We used a grid system of three differentially sized areas (A-, B-, and C- areas; see Section Materials and Methods) to analyse IPS-CM density and distribution following EB disaggregation (Figure 4A and Section Materials and Methods). After ~1 week in culture, all IPS-CM clusters were characterized by well-demarked troponin-T striation [Figure 4A(iii)] and there were no detectable time- or culture-dependent differences in cell shape, size or troponin-T distribution beyond this time. Entirely consistent with our finding that the disaggregation of older EBs was associated with poorer initial adherence of IPS-CMs to gelatin-coated glass (Figure 3C), we determined a weak negative correlation between the density of IPS-CM in A- and B- areas and the age of the EB at disaggregation (Figures 4B,C). Corroborating this finding, the plating heterogeneity, an index in which a larger number describes an increased heterogeneity of IPS-CM dispersal, increased markedly when IPS-CM were disaggregated from EBs > 2 weeks old (Figure 4D). The links apparent between the disaggregation of older EBs, reduced IPS-CM densities and the increased plating heterogeneity were also reinforced by the demonstration of a robust linear relationship between the sparseness of IPS-CM and the age of the EB at disaggregation (Figure 4E).

### Ca^2+^ signaling in IPS-CM clusters following EB disaggregation

**Figure 5:**
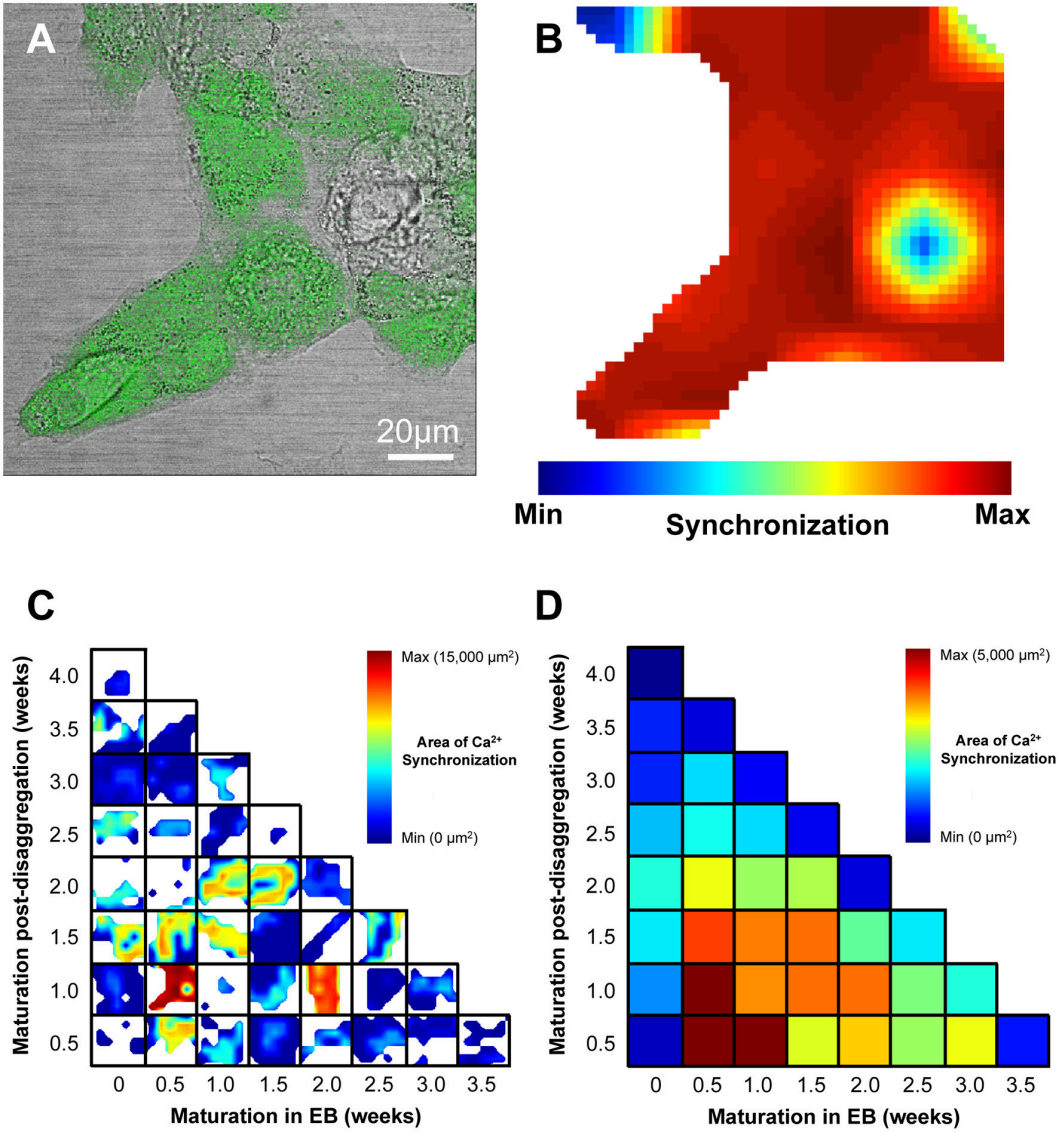
**Visualization of regional Ca^2+^ synchronization in IPS-CM clusters. (A)** A brightfield image of a typical IPS-CM cluster in a B-area overlaid with the corresponding fluo4-loaded image. **(B)** The regional Ca^2+^ synchronization map corresponding to the B-area of IPS-CM depicted in **(A)**. The map is generated from the temporal correlation of Ca^2+^ spikes across the B-area (0.021 mm^2^) following its sub-division into a 7 × 7 grid of C-areas (i.e., blue box, Figure 4A) as described in Section Materials and Methods. **(C)** Composite panel of regional Ca^2+^ synchronization in IPS-CM clusters in B-areas over the 4-week experimental matrix. The maximum Ca^2+^ synchronization determined under any condition was 34/49 C-areas (69.4%) which corresponded to a physical area of 14,571 μm^2^. White areas represent regions of the plate devoid of IPS-CM. **(D)** Color-coded visualization of the mean Ca^2+^ synchronization at each time point and for each condition across the 4-week experimental protocol (Figure 1B). Data are derived from *n* = 3 plates per time point.

We sought to determine whether the maturation of IPS-CM in EBs or “on plate” culture affected Ca^2+^ spike synchronization in IPS-CM clusters and focussed our analysis to the study of IPS-CM clusters in B-areas [arrowed, Figure 4A(ii)]. CLSM imaging of fluo4-dependent Ca^2+^ signaling in post-EB IPS-CM clusters (Figure 5A) was used to compile maps of regional Ca^2+^ synchronization across entire B-areas (Figure 5B). The plating of IPS-CM dissociated prior to any appreciable maturation phase in EB (i.e., maturation in EB = 0 weeks, vertical), or those from EBs > 2 weeks old, resulted in IPS-CM clusters that rarely supported spontaneous Ca^2+^ oscillations (Figures 5C,D). In contrast, the disaggregation of EBs between 0.5 to 1.5 weeks resulted in IPS-CM clusters characterized by high levels of spontaneous activity and regional Ca^2+^ synchronization (Figures 5C,D). A unifying feature of those IPS-CMs disaggregated from 0.5 to 1.5 week old EBs was the subsequent loss of spontaneous Ca^2+^ oscillations with continued “on plate” culture (Figures 5C,D, verticals).

**Figure 6:**
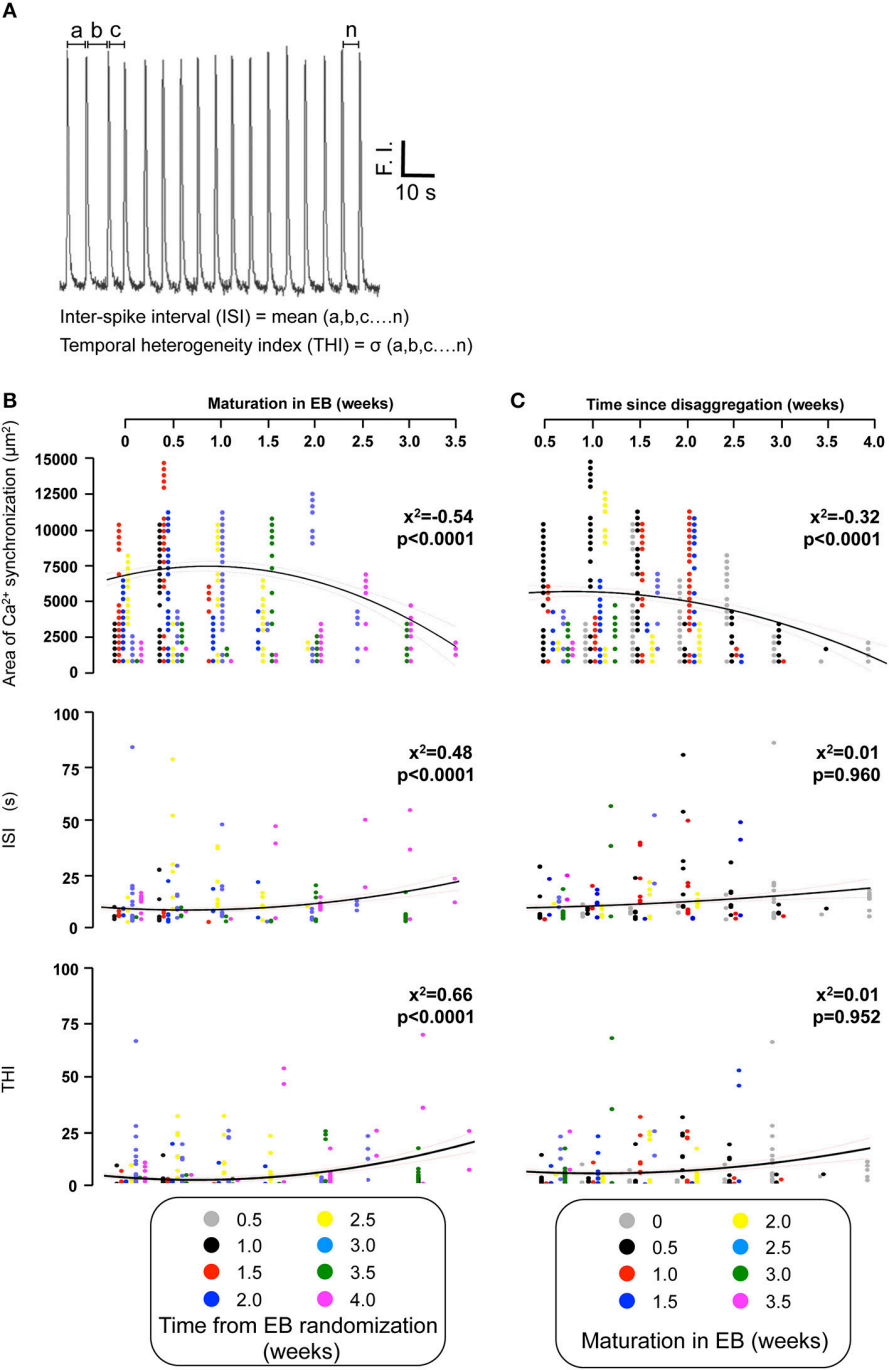
**Quantifying the temporal organization of Ca^2+^ spikes in spontaneously-oscillating IPS-CM clusters. (A)** The inter-spike interval (ISI) and temporal heterogeneity index (THI) was calculated from Ca^2+^ signal traces obtained from each C-area. F.I. is fluorescence intensity of fluo-4. **(B,C)** The effect of EB age **(B)** and the time of subsequent “on plate” culture **(C)** on regional Ca^2+^ synchronization (*upper panels*), ISI (*middle panels*), and THI (*lower panels*) in post-EB IPS-CM clusters is plotted. Data from different times points of the protocol are color coded as shown and were fitted with least-squares polynomial equations. The correlation co-efficients (*x*^2^) and *p*-values are given in each instance.

**Figure 7:**
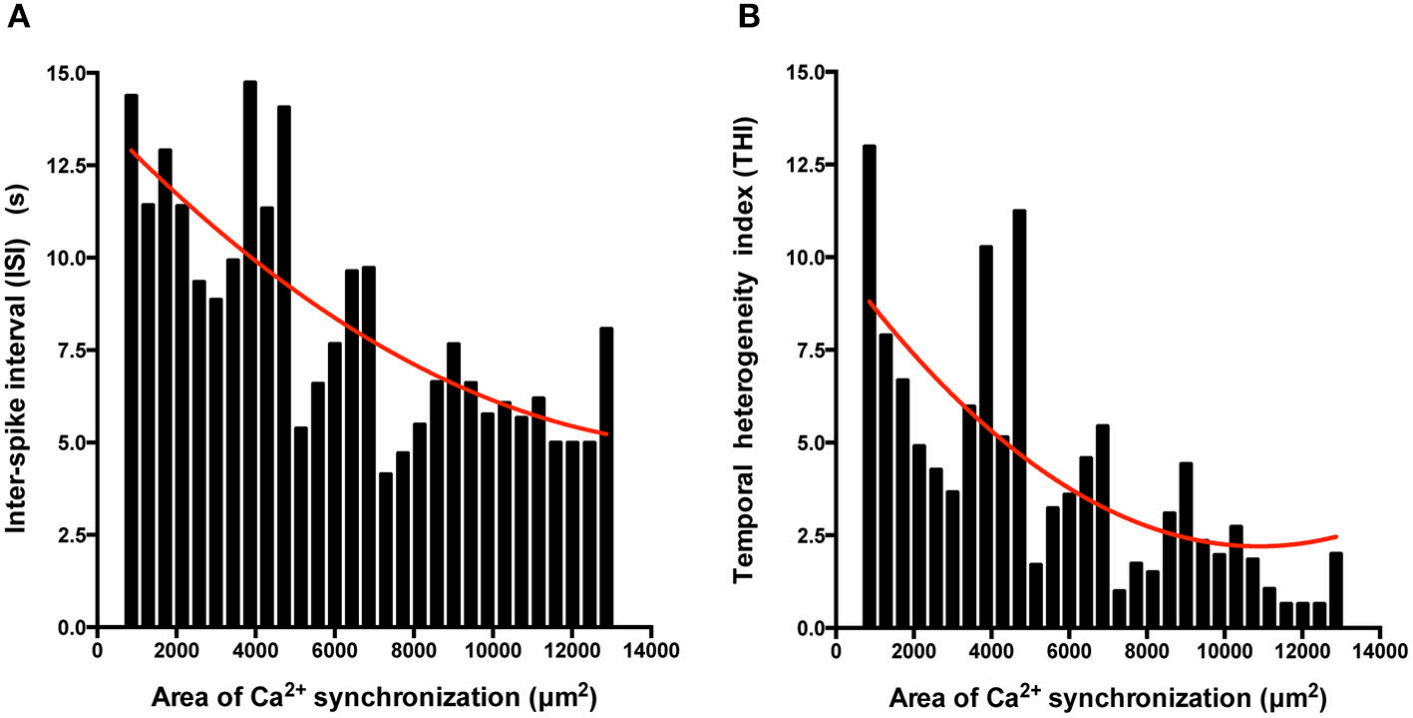
**The extent of regional Ca^2+^ synchronization is inversely correlated with ISI and THI. (A,B)** Histogram analysis of the relationship between ISI **(A)** and THI **(B)** and the extent of Ca^2+^ synchronization in post-EB IPS-CM clusters. Data were fitted with least-squares polynomial function (red line).

We also determined the temporal organization of Ca^2+^ spikes in the component C-areas using two indices, the inter-spike interval (ISI) and temporal heterogeneity index (THI) (Figure 6A), the latter increasing with increased temporal disordering of Ca^2+^ spikes in IPS-CM (Lewis et al., 2015). Consistent with the reduced regional Ca^2+^ synchronization in those IPS-CM clusters derived from EBs > 2 weeks old (Figures 5C,D, 6B, upper panel), ISI and THI were elevated in IPS-CM derived from these older EBs (Figure 6B, middle and lower panels, respectively). In contrast, although “on plate” culture was linked to progressive loss of regional Ca^2+^ synchronization in those IPS-CM exhibiting spontaneous activity on initial plating (Figures 5C,D, 6C, top panel), this phenomenon was not linked to the ISI and THI indices which remained unchanged over the time of IPS-CM “on plate” culture (Figure 6C, middle and lower panels, respectively). Taking all data together, there was a negative correlation between ISI and THI and the extent of regional Ca^2+^ synchronization (Figures 7A,B, respectively), suggesting that higher levels of regional Ca^2+^ synchronization were associated with lower ISI (Figure 7A) and THI values (Figure 7B; i.e., better organized Ca^2+^ spikes).

### IPS-CM quiescence and the differential effects of increased [Ca^2+^]_ext_

**Figure 8:**
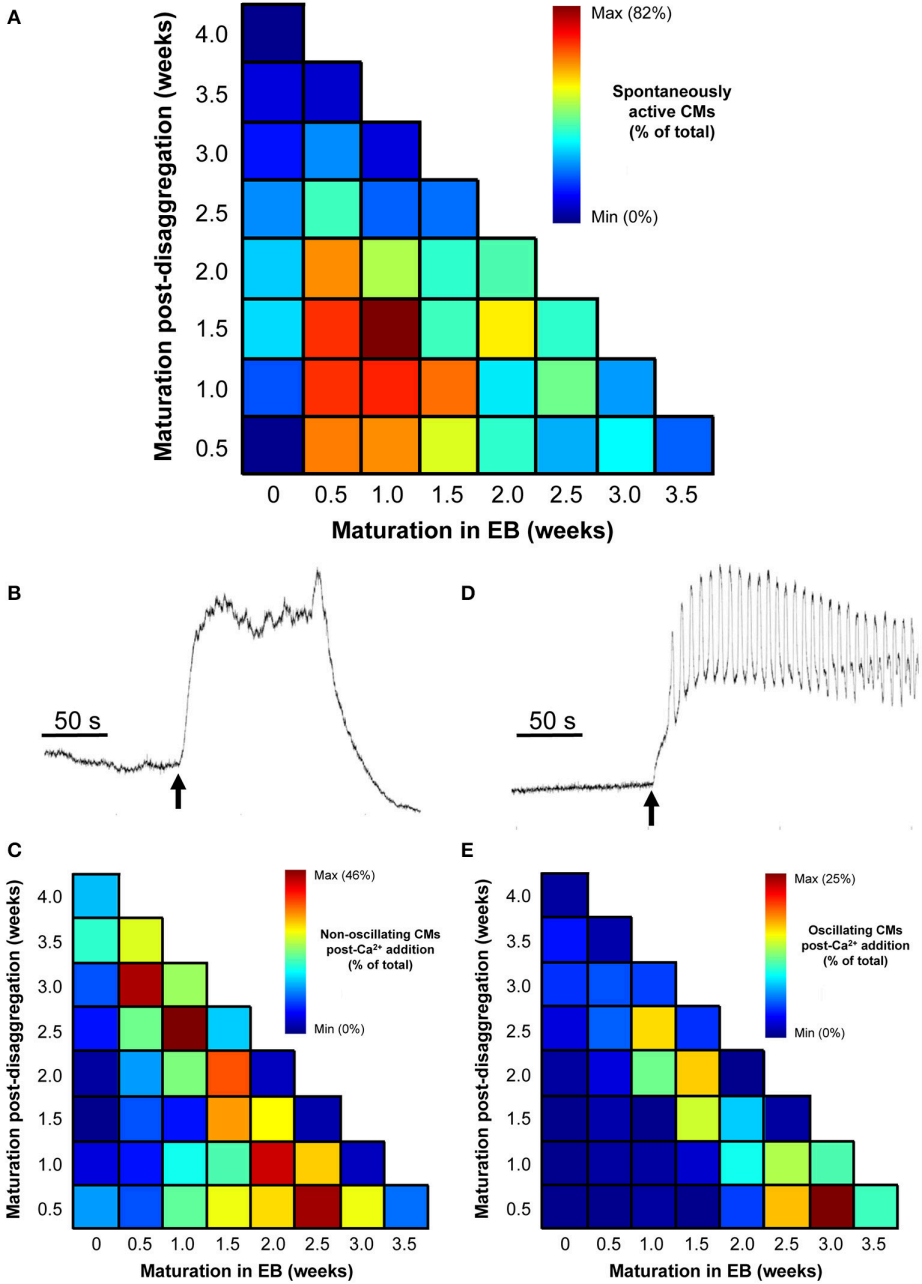
**Effect of increased [Ca^2+^]_ext_ in quiescent IPS-CMs. (A)** Color-coded visualization of the proportion of IPS-CMs that exhibited spontaneous Ca^2+^ oscillation following their disaggregation from EBs at different time periods (horizontal) or their transfer to “on plate” culture (vertical). **(B,C)** The proportion of IPS-CM across the experimental matrix that responded to increased [Ca^2+^]_ext_ by a sustained non-oscillatory elevation in [Ca^2+^]_cyt_ [typical of that shown in **(B)**] is visualized in **(C)**. **(D,E)** The proportion of IPS-CM across the experimental matrix that responded to increased [Ca^2+^]_ext_ by sustained [Ca^2+^]_cyt_ oscillations around an elevated [Ca^2+^]_cyt_ [typical of the pattern shown in **(D)**] is visualized in **(E)**. In **(C,E)** the mean data of *n* = 3 plates at each time point are given.

The proportion of spontaneously active post-EB IPS-CM clusters was dependent on the time of maturation of the IPS-CM within EBs with those typically dissociated from EBs between 0.5 and 1.5 weeks having the highest proportion of spontaneous Ca^2+^ spiking activity. Like the progressive reduction in the extent of regional Ca^2+^ synchronization in these IPS-CM clusters (Figures 5C,D), the reduced ability to support spontaneous Ca^2+^ spikes was a common feature of IPS-CM maintained “on plate” (Figure 8A). To explore this in more detail, we investigated the responsiveness of these IPS-CMs that had become non-oscillatory (“quiescent”) either as a result of “on plate” culture or those IPS-CM that typically did not exhibit inherent spontaneous Ca^2+^ spiking activity (i.e., those derived from EBs > 2 weeks old; Figures 5C,D, 8A) to raised extracellular Ca^2+^ ([Ca^2+^]_ext_). This protocol, in which [Ca^2+^]_ext_ was raised from ~0.4 mM to 1.8 mM, unmasked strikingly different responses in these quiescent IPS-CM. In some IPS-CM the increased [Ca^2+^]_ext_ triggered a large elevation in intracellular [Ca^2+^] which lasted for several minutes (Figures 8B,C). In others it provoked sustained high-amplitude Ca^2+^ oscillations around an elevated intracellular [Ca^2+^] (Figures 8D,E). The differential responses of IPS-CM clusters to increased [Ca^2+^]_ext_ mapped to whether their quiescence was inherent or culture-induced and were separable in the experimental matrix (Figure 8C vs. Figure 8E).

**Figure 9:**
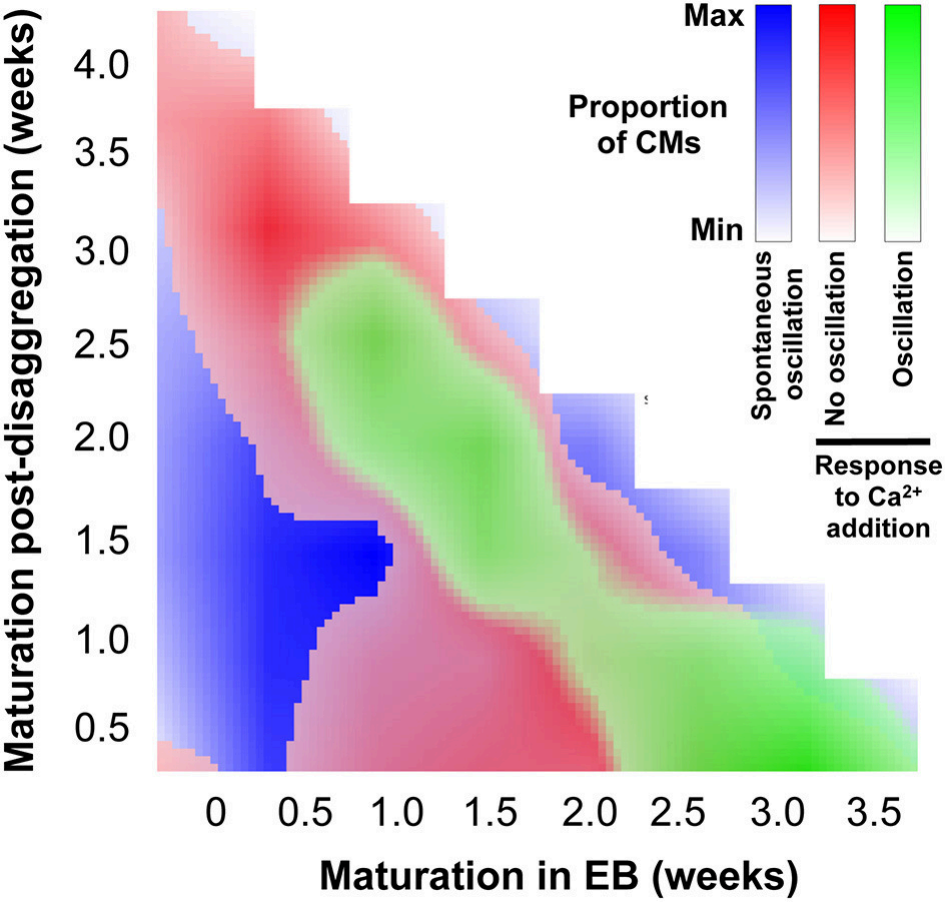
**Distribution of IPS-CM behavior across the experimental matrix**. A visual map that overlays the conditions that favor IPS-CM predisposition to spontaneous Ca^2+^ oscillatory behavior (blue; Figure 3), non-oscillatory behavior in response to increased [Ca^2+^]_ext_ (red; Figures 8B,C) and oscillatory behavior following increased [Ca^2+^]_ext_ (green; Figures 8D,E).

The data shown in Figure 8, together with the quantification of regional Ca^2+^ synchronization (Figures 5–7), were used to compile a distribution map of spontaneously-active Ca^2+^-synchronized IPS-CM and those IPS-CM clusters which exhibited innate or culture-dependent quiescence and which responded differently to increased [Ca^2+^]_ext_ (Figure 9).

## Discussion

We investigated the extent of regional Ca^2+^ synchronization in clusters of human IPS-CM that had been derived from EBs vs. those maintained in post-EB “on plate” culture over 4-weeks (Figure 1B). Intercellular Ca^2+^ synchronization across cell populations is collectively determined by multiple coupled oscillators acting across multiple scales (Novák and Tyson, 2008; Kholodenko et al., 2010; Kim et al., 2010a; Lakatta et al., 2010; George et al., 2012; Boileau et al., 2015) and thus the quantification of regional Ca^2+^ synchronization represents a robust index of IPS-CM functional competency. This study shows that tracking the changes in IPS-CM Ca^2+^ synchronization through two methods of maturation (EB vs. “on plate”) gives insights into some of the culture-dependent determinants that modulate IPS-CM Ca^2+^ handling behavior.

Cardiac cell signaling *in vivo* is modulated by interactions with neighboring cells and many approaches for directing cardiomyocyte maturation via the control of cell-to-cell communication have been developed (Tulloch et al., 2011; McSpadden et al., 2012; Tian and Morrisey, 2012; Zhang et al., 2012; Rao et al., 2013; Thavandiran et al., 2013; Howard and Baudino, 2014; Yang et al., 2014). An important factor in the present work therefore was the optimization of a gentle EB-disaggregation process that preserved the integrity of cell-to-cell contacts in the resulting IPS-CM clusters. These methods resulted in post-EB disaggregated IPS-CM clusters [arrowed, Figures 3A, 4A(ii)] that exhibited spontaneous rates of beating comparable to those of the source EBs.

The absence of regional Ca^2+^ synchronization (Figure 5D), spontaneous Ca^2+^ oscillation (Figure 8A) and the lack of response to elevated [Ca^2+^]_ext_ (Figures 8C,E) in those IPS-CM disaggregated from 0 week EBs are consistent with the view that CM maturation in the EB phase for even a very short time is a prerequisite for developing Ca^2+^-handling competency in IPS-CM populations.

IPS-CM clusters derived from EBs at 0.5–1.5 weeks exhibited the highest levels of regional Ca^2+^ synchronization (Figure 5D) and spontaneous Ca^2+^ oscillation activity (Figure 8A). The latter characteristic is a hallmark of immature cardiomyocyte phenotype and these data suggest that whilst 0.5–1.5 weeks in EB is sufficient to develop Ca^2+^ signaling competency, these IPS-CM are not functionally mature (adult-like) cardiomyocytes. Importantly, maintenance of these IPS-CM under “on plate” conditions was associated with the progressive elimination of regional Ca^2+^ synchronization (Figure 5D) and the loss of spontaneous Ca^2+^ oscillations (Figure 8A). There are numerous factors that potentially contribute to the cessation of spontaneous Ca^2+^ oscillations in IPS-CM obtained from 0.5 to 1.5 week EBs following “on plate” culture and this issue warrants further investigation. However, we can eliminate the influence of the low [Ca^2+^] environment. RPMI-based media containing relatively low [Ca^2+^] (~0.4 mM) was used throughout this study and the spontaneous contractility observed in EBs across the 4-week protocol reveals that 0.4 mM [Ca^2+^]_ext_ is fully compatible with the development and persistence of spontaneous Ca^2+^ oscillations. It is plausible however that the comparatively low extracellular Ca^2+^ environment used in Burridge's protocol (Burridge et al., 2011) does influence some aspects of EB development and function. IPS-CM morphology, shape, density and viability remained unchanged during “on plate” culture but it is possible that cell maintenance in this configuration for up to 3.5 weeks does alter the profile of atrial, nodal or ventricular-type activities within the IPS-CM population. A more in-depth exploration of this issue is needed. Since we have argued above that one of the key features marking the transition from immature to mature cardiomyocyte phenotype is the loss of spontaneous Ca^2+^ oscillatory behavior, we propose that “on plate” post-EB culture may be associated with the development of a more mature IPS-CM phenotype. This conclusion, which is well aligned with methodological developments aimed at monolayer-oriented protocols (van den Berg et al., 2016), and extends previous reports of CM maturation in EBs (Snir et al., 2003; Burridge et al., 2011), is supported by our demonstration of the re-emergence of well-ordered Ca^2+^ oscillations in these “on plate”-induced “Ca^2+^ quiescent” IPS-CM populations following an increase in [Ca^2+^]_ext_ and points to their comparatively mature Ca^2+^ handling machinery. It should be noted that the increased [Ca^2+^]_ext_ used in these experiments (~1.8 mM) does not represent a “physiologically” high Ca^2+^ environment ([Ca^2+^]_ext_ *in vivo* is around 1.3 mM; Bers, 2001) but it is substantially higher than the comparatively low [Ca^2+^]_ext_ that the EB and IPS-CM are maintained in (~0.4 mM).

At the molecular level, the development of a more mature ventricular-like cardiomyocyte phenotype during “on plate” culture is consistent with the loss of the components that promote automaticity (e.g., reduced expression of pace-making HCN (IK_f_, funny current) and T-type Ca^2+^ channels (Lakatta and DiFrancesco, 2009; Lakatta et al., 2010) and the possible re-balancing of “voltage clocks” and “Ca^2+^ clocks” (Weisbrod et al., 2013). This remains to be investigated in these IPS-CM clusters. We cannot exclude the possibility that a recovery of spontaneous Ca^2+^ oscillatory activity in these IPS-CM clusters occurs beyond the 4-week period of this study and thus a longer-term assessment of IPS-CM Ca^2+^ signaling is now warranted. The use of genetically encoded Ca^2+^ indicators (Kaestner et al., 2014) that enable the non-invasive tracking of Ca^2+^ signaling events over much longer time periods would facilitate such an approach.

In contrast to the spontaneous activities of IPS-CM derived from EBs between 0.5 and 1.5 weeks, the disaggregation of EBs older than 2 weeks resulted in a decreased number of adherent, spontaneously active IPS-CM (Figure 3). The physical dimensions and cellular occupancy of the source EBs remained comparatively constant over 4 weeks (Figure 2) and there was no evidence of cavitation or necrotic cores as has been reported by others (Gothard et al., 2009; Pettinato et al., 2014) (see Supplementary Movies 2–5). Furthermore, the decreased number of dissociated IPS-CM was not due to reduced post-EB cell proliferation since the extent of cell division following the disaggregation of all EBs was extremely low and we recorded just one cell division event during the entire study (Supplementary Movie 8). Thus, in our view, the most likely explanation for the decreased numbers of IPS-CM following disaggregation of later EBs (>2 weeks) is that changes in the nature of intercellular coupling via modulation of gap junction (connexin), tight-junction (ZO-1 and 2) and adherens junction proteins with EB maturation (Oyamada et al., 1996; Komura et al., 2008; Phua et al., 2014; Pettinato et al., 2015) limits the efficiency of IPS-CM dissociation from older EBs. The notion of enhanced cell-to-cell coupling in these older EBs is supported by the comparative functional “adult-like” maturity of the resulting IPS-CM clusters which typically lack spontaneous oscillatory behavior (Figure 8A) yet respond to elevated [Ca^2+^]_ext_ (Figures 8D,E).

Of relevance to arguments regarding the functional maturation of IPS-CM, it should be noted that all post-EB IPS-CM clusters were characterized by intracellular striation of troponin-T [Figure 4A(iii)]. Since IPS-CM studied in these investigations exhibited a broad range of Ca^2+^ handling synchronization and spontaneous activity across a 4-week period, our data would suggest that whilst troponin-T labeling remains an extensively used immunomarker of cardiomyocytes, it does not discriminate the maturity of the cardiomyocytes (Gorza et al., 1996; Sheng et al., 2012; Khan et al., 2013). This finding concurs with our previous data that showed that troponin-T immunoreactivity and striation *per se* is not a good index of the functional maturity of the Ca^2+^ handling machinery in human ES-derived cardiomyocytes (Lewis et al., 2015).

One of the most striking findings of these investigations is that IPS-CM devoid of the capacity to support spontaneous Ca^2+^ oscillations either innately or as a consequence of “on plate” culture responded differently to elevated [Ca^2+^]_ext_ (Figure 8). From these data, a picture thus emerges in which similar IPS-CM phenotypes (e.g., comparable Ca^2+^ handling, morphologies, densities and ages) underscored by different modes of derivation (e.g., EB vs. “on plate”) can result in very different response to external cues (e.g., elevated [Ca^2+^]_ext_). Spontaneous and Ca^2+^-triggered behavior in IPS-CM clusters can be traced back to their route of derivation and therefore a major finding of this study is that adaptive changes in Ca^2+^-signal organization under different maturation conditions are determinants of IPS-CM behavior. This finding is entirely in agreement with reports that cell function and fate may be imprinted via early-stage genetic and epigenetic events (Beqqali et al., 2006; Kim et al., 2010b; Balázsi et al., 2011; Li et al., 2012; Cahan et al., 2014; Morris et al., 2014). Our data also support the intriguing possibility that IPS-CM responses can be predicted from a detailed knowledge of their routes of derivation and maturation. In this proof-of-concept study, we focussed on the analysis of Ca^2+^ signal organization as a means to systematically track the functional maturation of Ca^2+^ synchronization in IPS-CM following in EBs vs. “on plate” culture. Further work is currently underway to determine whether such an approach can be utilized to explore other endpoints including ion channel expression profile, selective responses to drugs (e.g., to β-adrenoceptor antagonists, Ca^2+^-sensitizers) and IPS-CM tolerance to massive scale culturing required to generate the vast numbers of cells required for heterologous and autologous cellular transplantation in new heart repair approaches (Mummery et al., 2010; Khan et al., 2013).

## Funding

This work was funded by the British Heart Foundation (FS/09/028/27602; RG/09/009/28069; CH/06/002/21631), Wellcome Trust (094219/Z/10/Z), and the Wales Deanery (School of Postgraduate Medical and Dental Education).

### Conflict of interest statement

The authors declare that the research was conducted in the absence of any commercial or financial relationships that could be construed as a potential conflict of interest.
