# Supplementary material for: A Systemized Approach to Investigate Ca2+ Synchronization in Clusters of Human Induced Pluripotent Stem-Cell Derived Cardiomyocytes
Source: Front Cell Dev Biol. 2016 Jan 13;3:89. doi: 10.3389/fcell.2015.00089 (PMC4710702; doi:10.3389/fcell.2015.00089)
Supplement: Supplementary file 9 [file DataSheet1.docx]

**Supplementary information**

**Supplementary Movie 1.** *EBs exhibit spontaneous and synchronized Ca^2+^ release.*

The movie is of an EB loaded with fluo4-AM (5µM, 30mins at 30°C followed by dye de-esterification for 30mins at 37°C) with 1,000 images acquired at 25 frames per second. The frequency of oscillation is 0.18Hz. In this example, high-amplitude Ca^2+^ release events, which occur throughout the entire EB, appear to be preceded by the automaticity of a small cluster of cells at the top left of the EB. The data evidence innate ‘pace-making’ within EBs.

**Supplementary Movie 2**. *Reconstructed CLSM z-stack from an EB at 0 weeks*

**Supplementary Movie 3.** *Reconstructed CLSM z-stack from an EB at 1 week*

**Supplementary Movie 4.** *Reconstructed CLSM z-stack from an EB at 2 weeks*

**Supplementary Movie 5.** *Reconstructed CLSM z-stack from an EB at 3 weeks*

In Supplementary Movies 2-5, in order to improve the visualization of the cellular arrangement in these comparatively flat EBs, the z-axis has been expanded by a factor of 4.

**Supplementary Movie 6.** *Time-lapse recording of cell populations after disaggregating 0 week EB.*

**Supplementary Movie 7.** *Time-lapse recording of cell populations after disaggregating 3 week EB.*

**Supplementary Movie 8.** *Cell division in post-disaggregated cell populations.*

This movie shows cell division following the disaggregation of a 0 week EB. This is the only recorded example of post- EB disaggregation cell division across the entire study.
